# Population Pharmacokinetics and Pharmacodynamics of Sitafloxacin in Plasma and Alveolar Epithelial Lining Fluid of Critically Ill Thai Patients With Pneumonia

**DOI:** 10.1002/prp2.70081

**Published:** 2025-03-23

**Authors:** Taniya Paiboonvong, Preecha Montakantikul, Navarat Panjasawatwong, Noppaket Singkham, Baralee Punyawudho

**Affiliations:** ^1^ Department of Pharmacy Practice College of Pharmacy, Rangsit University Pathum Thani Thailand; ^2^ Department of Pharmacy Faculty of Pharmacy, Mahidol University Bangkok Thailand; ^3^ Department of Pharmaceutical Care Faculty of Pharmacy, Payap University Chiang Mai Thailand; ^4^ Division of Clinical Pharmacy, Department of Pharmaceutical Care School of Pharmaceutical Sciences, University of Phayao Phayao Thailand; ^5^ Department of Pharmaceutical Care Faculty of Pharmacy, Chiang Mai University Chiang Mai Thailand

**Keywords:** alveolar epithelial lining fluid, critically ill patients, Monte Carlo simulations, population pharmacokinetics, Sitafloxacin

## Abstract

Sitafloxacin is one of the oral respiratory quinolones for the treatment of community‐acquired pneumonia. The pharmacokinetic (PK) changes of sitafloxacin in critical illness have been previously reported. However, sitafloxacin exposure and target attainment have never been confirmed in this population. To develop a population pharmacokinetic (PK) model of sitafloxacin, plasma and epithelial lining fluid (ELF) concentrations were obtained after sitafloxacin administration as a 200‐mg single dose under fasting condition in 12 subjects. A population pharmacokinetic analysis was performed using a nonlinear mixed‐effects modeling approach. The probability of target attainment (PTA) and cumulative fraction of response (CFR) against the MIC distribution of *S. pneumoniae* isolated from Thai patients was estimated by Monte Carlo simulations. The pharmacokinetics of sitafloxacin in plasma was best described by a one‐compartment model linking to the ELF compartment. The partition coefficient which relates drug exposure in ELF to drug exposure in plasma was estimated to be 0.77. Age was a significant covariate that impacted the relative bioavailability. Results from Monte Carlo simulations showed that the maximum approved dose of sitafloxacin 100 mg q 12 h provided > 90% PTA and CFR in both plasma and ELF. The current maximal dosing of sitafloxacin provided adequate exposure in plasma and ELF for the treatment of critically ill Thai patients with pneumonia.

## Graphical abstract

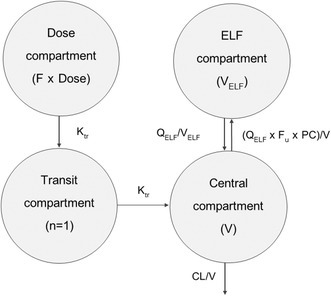

## 1 Introduction

Severe community‐acquired pneumonia (CAP) requires admission to an intensive care unit and is associated with high mortality. *Streptococcus pneumoniae* continues to be the leading cause of bacterial pneumonia, although Thailand has documented rising prevalence rates of beta‐lactam and macrolide‐resistant *S. pneumoniae* [1]. Sitafloxacin is a broad‐spectrum fluoroquinolone that shows potent activity against drug‐resistant *S. pneumoniae* , including strains resistant to other fluoroquinolones. The oral formulation of sitafloxacin has been approved and used for the treatment of respiratory tract infections in Thailand since 2012, with a recommended dose of 50–100 mg twice daily. It also shows good oral absorption with excellent bioavailability and tissue distribution in pneumonia patients [2]. Similar to other fluoroquinolones, sitafloxacin exhibits concentration‐dependent killing, and the area under the plasma concentration–time curve (AUC) to the MIC is considered the pharmacokinetic/pharmacodynamic (PK/PD) parameter best predicting efficacy [3]. The 24‐h free‐drug AUC/MIC (*f*AUC/MIC) > 30 is considered the PK/PD target according to a previous clinical dose‐finding study [4].

Alveolar compartments, such as epithelial lining fluid (ELF), are considered an important source of infection in patients with pneumonia; hence, sufficient antibacterial concentration is necessary in this area. Critically ill patients exhibit a number of physiological alterations that can alter the PK of antibiotics, particularly beta‐lactam antibiotics [5, 6]. Although the alteration of PK in critically ill patients has previously been observed for fluoroquinolones [3], limited information has been presented regarding the PK of sitafloxacin in plasma and ELF in critically ill patients. Even though the PK of sitafloxacin in critically ill Thai patients has been investigated [7], the impact of patient characteristics and other important factors on sitafloxacin pharmacokinetics has never been investigated. Additionally, the optimal dosage regimens to achieve the PK/PD target for this group of patients have never been explored. Therefore, this study aimed to develop a population pharmacokinetic model of sitafloxacin in plasma and ELF. Model‐based simulations were then applied to investigate the optimal dosage regimens of sitafloxacin achieving target PK/PD value.

## 2 Methods

### 2.1 Patient and Pharmacokinetic Sampling

Data from the previous pharmacokinetic study of sitafloxacin were used in this analysis. Study design, subjects, and analytical methods were described previously [7]. In brief, all subjects received a single oral dose of sitafloxacin 200 mg under fasting conditions. Blood samples were collected at pre‐dose, 0.5, 1, 2, 3, 8, and 12 h. Additionally, one bronchoalveolar lavage (BAL) sample was randomly collected at different time intervals: 0.5–2, 3–4, 5–6, and 7–9 h for each patient. Sitafloxacin concentrations in plasma and ELF were measured using LC–MS/MS. The lower limit of quantification (LLOQ) was 0.025 mg/L, considered the lowest measured concentration with an accuracy and precision of ± 20%.

### 2.2 Population Pharmacokinetic Analysis

**FIGURE 1:**
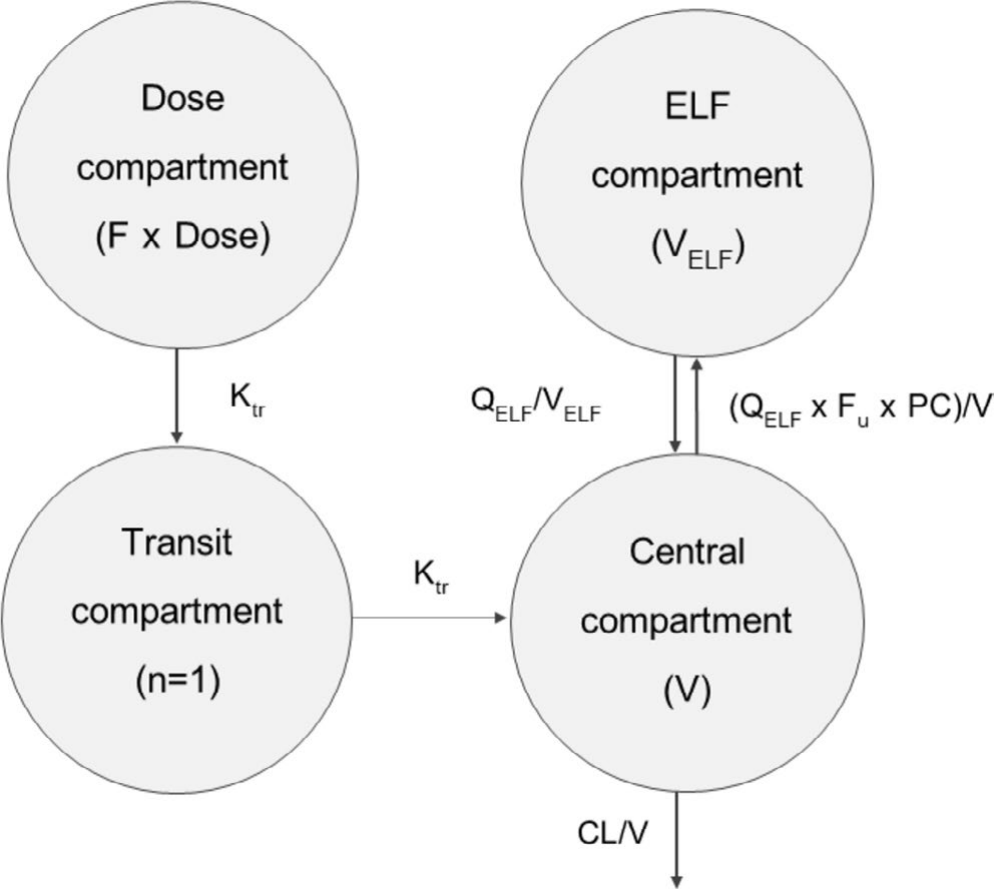
Schematic diagram of the final PK model for sitafloxacin after oral administration.

The population PK analysis was conducted using a nonlinear mixed‐effects model (NONMEM version 7.4; Icon Development Solution, Ellicott City, MD). The first‐order conditional estimation with eta‐epsilon interaction (FOCEI) was applied throughout model development. A NONMEM code of the final model was provided in Data S1. Post‐processing of NONMEM outputs and graphical diagnostics were performed using R version 3.4.4 (R Development Core Team; www.r‐project.org), Xpose version 4.5.3, and Perl‐speaks‐NONMEM (PsN) version 4.7.0. The plasma and ELF sitafloxacin concentrations were converted into a natural logarithm. One plasma concentration was below the lower limit of quantification and was excluded from the data. For the structural model development, one‐, two‐, and three‐compartment models were tested to fit the plasma data. Several absorption models, that is, first‐order absorption (with or without lag time) and transit compartment models, were tested [8]. Relative oral bioavailability (F) was fixed to 1, and its inter‐individual variability (IIV) was estimated. An ELF compartment was added to the developed structural model as one of the peripheral compartments (Figure 1).

The IIV was modeled exponentially, resulting in log‐normally distributed parameters. The additive, proportional, or combined error models were tested to describe the residual variability. Structural model selection was guided by the decrease in objective function value (OFV = −2 log‐likelihood) for nested models, the Akaike information criteria (AIC) for non‐nested models, the relative standard errors (RSEs) of the parameter estimates, and the goodness‐of‐fit (GOF) plots. Body weight was added as an allometric function a priori on CL/F with the exponent fixed to 0.75 and volume of distribution (V/F) with the exponent fixed to 1. The covariates, including age, sex, and creatinine clearance (CLcr), estimated by the Cockcroft and Gault equation and the acute physiology and chronic health evaluation (APACHE) II score were screened for their significant impact on the PK parameters using a stepwise procedure. The difference in OFV (ΔOFV) > 3.84 (*χ* ^2^, df = 1, *p* < 0.05) and 6.63 (*χ* ^2^, df = 1, *p* < 0.01) was used as cut‐off criteria for forward selection and backward deletion, respectively.

### 2.3 Model Evaluation

The final PK model was evaluated using sampling importance resampling (SIR) and visual predictive check (VPC). Three iterations with the number of SIR samples (M) and resamples (m) of 1000 M and 200, 400, and 500 m were performed to estimate the uncertainties of the final model parameters [9]. The parameter values of the final model derived using NONMEM were compared with the median and 95% CI (2.5th and 97.5th percentiles) of the parameter estimates derived from SIR. Visual predictive checks as simulation‐based diagnostics were performed to assess the predictive variability of the final model. The final PK model was used to simulate 1000 concentrations. The 95% confidence intervals (95% CIs) were constructed from simulated concentration–time profiles and plotted against the 5th, median, and 95th percentiles of the observed concentrations.

### 2.4 Monte Carlo Simulation

Monte Carlo simulations were performed using the final model to predict steady‐state AUC_0‐24_ in plasma and ELF for 5000 virtual adults with ages of 30, 40, 50, 60, and 70 years. The probability of achieving the PK/PD target for a range of clinically relevant MIC values (0.032–0.25 mg/L) was explored [10] when the current dosage regimens approved for the treatment of pneumonia (50 mg q 12 h and 100 mg q 12 h) were administered. Sitafloxacin concentration time profiles in plasma and ELF at steady state were simulated for 5000 virtual patients. The *f*AUC of sitafloxacin at steady state was estimated based on a fraction unbound of 63% [7]. The percentage of target attainment (PTA) and cumulative fraction of response (CFR) were calculated. The PTA for achieving the target of *f*AUC/MIC > 30 for each dosing regimen was estimated for each individual MIC among the isolates. The CFR was calculated as the population PTA predicted for a given drug dose and MIC distribution. The PTA and CFR ≥ 90% were considered as probabilities of success. The susceptibility data utilized in this study were based on the clinical isolates of *S. pneumoniae* obtained from Thai patients [10].

## 3 Results

### 3.1 Characteristics of Patients

There were a total of 12 patients included in this study. Of the 12 patients, six were male. The median (IQR) age, weight, and CLcr were 57 (40–65) years, 52 (44–68) kg, and 68 (30–96) mL/min, respectively. The median APACHE II score was 21 (IQR: 18–33) and the median albumin was 2.0 (IQR: 1.8–2.2) g/dL.

### 3.2 Microbiology

The MIC range of sitafloxacin against the clinical isolates was 0.032–0.25 mg/L. The MIC50 and MIC90 values were identical (0.0625 mg/L). Table 1 presents the MIC distribution and percentage of all isolates.

**TABLE 1:** Cumulative percentage of the isolates at the MIC (mg/L).

MIC (mg/L)	Cumulative percentage
0.032	40
0.0625	97
0.125	99
0.25	100

### 3.3 Population PK Analysis

The pharmacokinetic model of sitafloxacin was developed using 83 plasma and 12 ELF concentrations from 12 patients. One plasma concentration was below the limit of quantification and was excluded from the dataset. A one‐compartmental model best described the pharmacokinetics of sitafloxacin. No further improvement was seen with a two‐compartment model (ΔOFV = 0, ΔAIC = +4.0). The inclusion of one fixed transit absorption compartment, which set the absorption rate constant (Ka) to be identical to the transfer rate constant (Ktr), improved the model fit. (ΔOFV = −19.78, ΔAIC = −19.78) . When the ELF compartment was incorporated, the partition coefficient (PC) for distribution into the ELF compartment was estimated to be 77.2%. As the volume of distribution of the ELF (V/F_ELF_) could not be reliably estimated, the V/F_ELF_ was fixed at a value of 0.025 L, which was obtained from a previous physiological study of the lung model [11]. Moreover, the data did not support the estimation of IIV of V/F and intercompartmental clearance of ELF (Q/F_ELF_); thus, V/F and Q/F_ELF_ were estimated without their IIV. An additive residual error model on the logarithmic scale was used to describe the residual variability (RUV) for both plasma and ELF concentrations.

**FIGURE 2:**
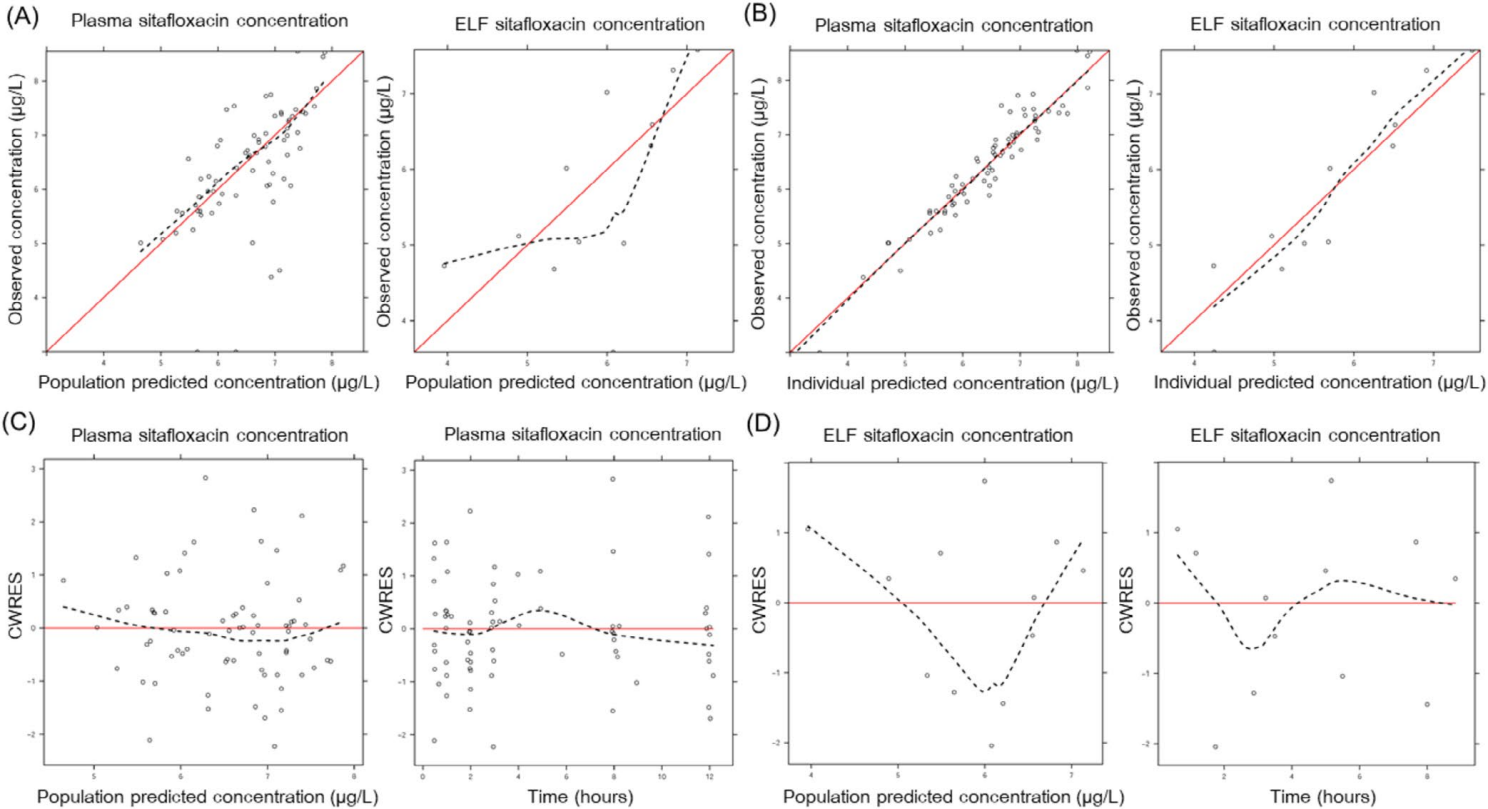
Goodness‐of‐fit plots for the final model. (A) Observed data (DV) versus predictions (PRED) in plasma and ELF. (B) Observed data (DV) versus individual predictions (IPRED). (C) Conditional weighted residual errors (CWRES) versus population predicted concentration and time after dose in plasma. (D) Conditional weighted residual errors (CWRES) versus population predicted concentration and time after dose in ELF.

**FIGURE 3:**
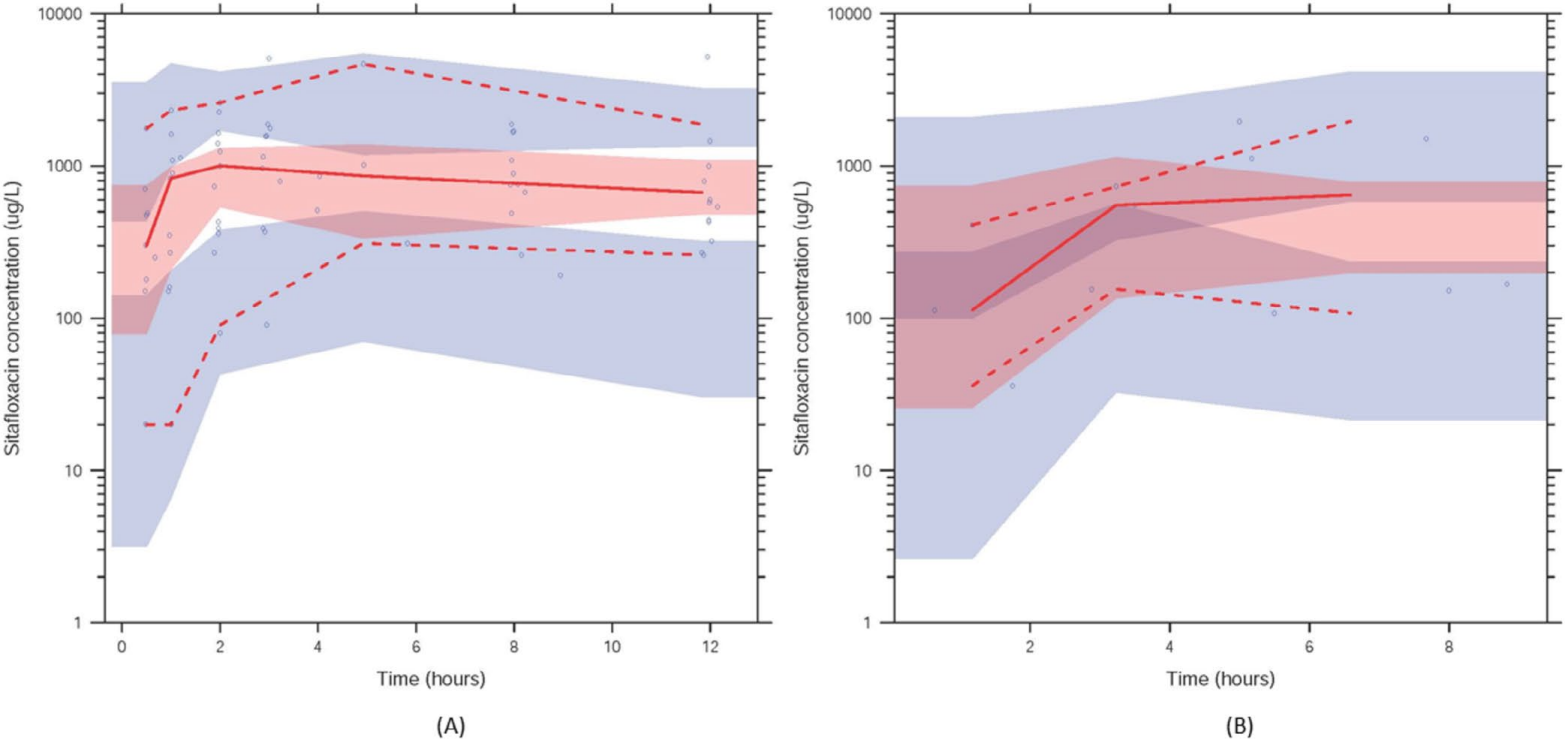
Visual predictive check plot of the final model. (A) Plasma. (B) ELF.

Among tested covariates, age was a statistically significant covariate on bioavailability (F). The relationship between age and F was explained by a linear function (F = 1+ (Age‐57) * 0.0258). Every 1‐year increase in age resulted in a 2.58% increase in F. The parameter estimates of the final model and results from the SIR analysis are presented in Table 2. The goodness‐of‐fit plots demonstrated that the final model described the data adequately (Figure 2). The visual predictive check plots of plasma and ELF showed that the median, 5th, and 95th percentiles of the observed data were overlaid with the corresponding percentiles of the simulated data (Figure 3). Therefore, the final model's predictive performance is acceptable.

**TABLE 2:** Population PK parameter estimates for the final model.

Parameters	Population estimate^a^ (%RSE)^b^	95%CI^b^	%CV for IIV^a^ (%RSE)^b^	95%CI for IIV^b^
F	1 *fix*	—	17.0 (17.1)	9.87–22.1
CL/F (L/h/52 kg)	7.03 (24.9)	4.36–11.0	87.2 (17.8)	45.9–125
V/F (L/52 kg)	116 (13.0)	93.9–153	—	—
MTT (hr)	1.48 (27.1)	0.858–2.44	93.3 (27.1)	64.7–177
V/F_ELF_ (L)	0.025 *fix*	—	—	—
Q/F_ELF_ (L/h/52 kg)	0.0441 (36.5)	0.0194–0.0812	—	—
f_u_	0.63 *fix*	—	—	—
PC	0.772 (18.2)	0.556–1.08	23.3 (18.4)	17.2–33.0
AGE on F (%)	2.58 (7.64)	2.09–2.84	—	—
*σ*_Plasma_	36.0 (10.5)	29.4–44.6	—	—
*σ*_ELF_	53.2 (14.4)	36.3–70.8	—	—

Abbreviations: *σ*, variance of the residual variability, incorporated as an additive error on the logarithmic scale; CL/F, oral elimination clearance; ELF, epithelial lining fluid; F, relative bioavailability; f_u_, fraction unbound; IIV, inter‐individual variability; MTT, mean transit absorption time; PC, partition coefficient between central and ELF compartment; Q/F_ELF_, inter‐compartmental clearance between central and ELF compartment; V/F, volume of distribution of central compartment; V/F_ELF_, volume of distribution of the ELF compartment.

^a^ Population mean parameter estimates from NONMEM.

^b^ Assessed by sampling importance resampling (SIR).

### 3.4 Monte Carlo Simulation

Given the limited extent of change in the predicted *f*AUC_0‐24_ across age groups, the simulated *f*AUC_0‐24_ for all groups was combined and shown. The simulation results showed that the median *f*AUC_0‐24_ in plasma and ELF following administration of 100 mg of sitafloxacin q 12 h was 21.02 μg·h/mL and 10.17 μg·h/mL, respectively. The median *f*AUC_0‐24_ in plasma and ELF for a dosing schedule of 50 mg q 12 h was 10.42 μg·h/mL and 5.12 μg·h/mL, respectively. The PTA of sitafloxacin 100 mg q 12 h was > 90% for MIC ≤ 0.125 mg/L in plasma and MIC ≤ 0.0625 mg/L in ELF. For sitafloxacin 50 mg q12 h, the PTA was > 90% for MIC ≤ 0.0625 mg/L in plasma and MIC ≤ 0.032 mg/L in ELF. When sitafloxacin 100 mg q 12 h was simulated, the CRF in plasma and ELF were expected to be 99.80% and 95.96%, respectively. While the CRF was 96.97% in plasma for sitafloxacin 50 mg q 12 h, the CRF of ELF was 87.96%. Table 3 presents the %PTA of sitafloxacin 100 mg q 12 h and 50 mg q 12 h for each MIC value in plasma and ELF.

**TABLE 3:** The probability of target attainment (PTA) of each dosage regimen in plasma and ELF (PK/PD index *f*AUC/MIC ≥ 30).

MIC (mg/L)	No. of isolates	STX 100 mg q 12 h		STX 50 mg q 12 h	
		Plasma	ELF	Plasma	ELF
0.032	40	100.00	99.18	100.00	95.34
0.0625^a^	57	100.00	95.36	96.34	84.84
0.125	2	94.60	79.44	81.42	55.70
0.25	1	85.72	62.50	63.48	33.70

^a^ MIC50/90 = 0.0625/0.0625 mg/L.

## 4 Discussion

In this study, a population PK model was developed to characterize plasma and ELF concentrations of sitafloxacin in critically ill patients. This is the first population PK modeling of sitafloxacin in critically ill patients in which both plasma and ELF population PK parameters were established. Furthermore, the probability of achieving the PK/PD target for various MIC values of sitafloxacin in plasma and ELF was examined using Monte Carlo simulation. A one‐compartment model best described the PK of sitafloxacin in plasma, which is consistent with the results from a previous population PK study of sitafloxacin in noncritically ill Japanese patients [12].

Significant physiological changes are common among critically ill patients. These pharmacokinetic changes may result in decreased achievement of PK/PD targets. Gastrointestinal dysmotility frequently presents in critical illness, leading to delayed absorption of several drugs [13]. Thus, models were tested to describe the delayed absorption of sitafloxacin in this population. Among all the delayed absorption models tested, a transit model best described the data and resulted in an estimated MTT, an average time to transit to the absorption compartment, of 1.48 h with the IIV of 93.3%. A high IIV of MTT reflects substantial variability in the delayed absorption in this population.

In our study, the estimated CL/F and V/F of sitafloxacin were 7.03 L/h and 116 L in patients weighing 52 kg, which were higher than the values previously reported in non‐critically ill patients [12]. The estimated CL/F from our investigation was marginally lower than what was reported from a previous study (7.03 L/h vs. 8.82 L/h). Given that our patients were critically ill, diminished renal function, a primary pathway for sitafloxacin elimination was to be expected. A higher V/F may be attributable to hypoalbuminemia, which is common in critically ill patients. With a higher V/F, a higher loading dose of sitafloxacin in order to achieve the PK/PD target may be required in this population.

According to the physiochemical properties of quinolones, they are capable of penetrating the respiratory tract, particularly the ELF and alveolar cells [14]. However, there was variation in permeability among populations, which may be linked to disease diversity or severity of lung inflammation [15, 16, 17]. In our study, the PC of sitafloxacin to ELF was estimated to be 0.77 with good precision (RSE 18%). As approximately 80% of the plasma concentration of sitafloxacin can be diffused into ELF, this may indicate that sitafloxacin is well diffused into ELF in critically ill patients.

Age‐related changes in physiology could impact a drug's pharmacokinetics. The results from this study found age was a significant covariate for bioavailability. Age‐related increases in bioavailability were observed. Sitafloxacin is a basic drug, thus increasing stomach pH in advancing age may increase unionized form of sitafloxacin leading to an increase in absorption. In addition, since aging may be associated with a decrease in first‐pass metabolism, bioavailability may increase [18]. Creatinine clearance has been demonstrated to impact CL/F of sitafloxacin [12]. Surprisingly, the effect of CLcr was not observed in our study. This may be related to the limited range of renal function in this study, as the majority of participants had mild to moderate renal impairment (66.7%). It is noteworthy that one patient in our study shows a creatinine clearance (CLcr) of 235 mL/min, indicating of augmented renal clearance, which may be classified as an outlier. Augmented renal clearance is a phenomenon commonly encountered in critically ill patients. In these patients, dose adjustment is essential to prevent subtherapeutic levels. Unfortunately, our dataset had a limited number of patients, and CLcr was not recognized as a significant covariate in the final model. Further research including a larger cohort of patients with varied renal function is essential to determine the optimal dose adjustment according to patients' renal function.

A Monte Carlo simulation was performed to evaluate the percentage of target attainment of sitafloxacin in critically ill patients with pneumonia. The simulation results showed that sitafloxacin 100 mg q 12 h can achieve the PTA and CFR ≥ 90% in both plasma and ELF for MIC ≤ 0.0625 mg/L. However, only the plasma target can be reached when sitafloxacin 50 mg q 12 h was administered. Although the ELF target was not reached with the dose of 50 mg q 12 h for MIC ≥ 0.0625 mg/L, high PTA and CFR (> 80%) were observed. Therefore, this regimen may be applicable to use for the treatment of CAP caused by *S. pneumoniae* . Interestingly, our study revealed that isolates with MIC > 0.625 mg/L may require a higher dose than 100 mg q 12 h to achieve the target in ELF. Although the maximum recommended dose of sitafloxacin in Japan and Thailand is 100 mg q 12 h, the use of higher doses has been reported, and the safety and tolerability of intravenous sitafloxacin 400 mg once daily have been proven [19, 20, 21].

The susceptibility breakpoint of sitafloxacin has never been reported by the Clinical and Laboratory Standards Institute (CLSI) or the European Committee on Antimicrobial Susceptibility Testing (EUCAST) [22, 23]. Yamagishi Y et al. [24] proposed breakpoints of 0.0625 mg/L for the dose of 50 mg q 12 h and 0.125 mg/L for the dose of 100 mg q 12 h. According to the results from our study, the proposed breakpoints of sitafloxacin against *S. pneumoniae* based on %PTA in ELF are 0.0625 mg/L for the 100 mg q 12 h dose and 0.032 mg/L for the 50 mg q 12 h dose.

Our study has some limitations. Due to the small number of patients with sparse ELF samples included in the population PK study, it was not possible to determine the IIV of some PK parameters, including V/F, V/F_ELF_, and Q/F_ELF_. A previous study showed the influence of ABCB1, UGT1A1, and UGT1A9 polymorphisms on the pharmacokinetics of sitafloxacin. However, the impact of these gene polymorphisms on sitafloxacin pharmacokinetics was not examined in our study. In addition, the isolates were collected from a tertiary university hospital in Bangkok, which may differ from other settings and influence the CFR‐based PD analysis. Our findings, however, are applicable to specific MIC pathogens. In summary, the population PK of sitafloxacin in plasma and ELF was successfully developed. The results from this study confirmed that the current maximum recommended dose of sitafloxacin, 100 mg q 12 h, can achieve the PTA and CFR > 90% in both plasma and ELF. Thus, this dosage regimen is deemed optimal for treating *S. pneumoniae* pneumonia in the ICU.

## Author Contributions

T.P. and B.P. wrote and revised the manuscript. N.P. wrote some parts of the manuscript. T.P., P.M., and B.P. contributed to the conceptualization and design of the study. T.P., P.M., N.P., N.S., and B.P. performed the analysis and interpretation of the data. All authors read and approved the final manuscript.

## Ethics Statement

This study was approved by the Institutional Review Board of the Faculty of Dentistry/Faculty of Pharmacy, Mahidol University, Bangkok, Thailand (MU‐DT/PY‐IRB 2019/052.0309).

## Conflicts of Interest

The authors declare no conflicts of interest.

## Supporting information


Data S1.

## Data Availability

The datasets used and/or analyzed for this study are available from the corresponding author upon request.
