# Supplementary material for: Population Pharmacokinetics and Pharmacodynamics of Sitafloxacin in Plasma and Alveolar Epithelial Lining Fluid of Critically Ill Thai Patients With Pneumonia
Source: Pharmacol Res Perspect. 2025 Mar 23;13(2):e70081. doi: 10.1002/prp2.70081 (PMC11930543; doi:10.1002/prp2.70081)
Supplement: Supplementary file 1 — Data S1. [file PRP2-13-e70081-s001.docx]

**Supplementary**

**Final model code**

**$PROBLEM** Run56

;; 1-compartment disposition

;; 1 transit compartment (KA=KTR)

;; Add allometric function on QELF

;; Body weight as allometric function (median body weight)

**$INPUT** ID ; Patient ID

TIME ; Hours

DV1 = DROP ; Conc. (ug/L)

DV ; Natural logarithm conc. (ug/L)

AMT ; Sitafloxacin dose (ug)

MDV ; Missing dependent variable: MDV=0 (DV); or MDV=1 (Dose)

EVID ; Event ID 0=Sample; Dose, 1= Dose

CMT ; Compartment (1=Depot, 2=Blood, 3=ELF)

AGE ; Age (year)

SEX ; Sex (0=male,1=female)

WT ; Body weight (kg)

CLCR ; Creatinine clearance (mL/min)

APACHE ; Acute physiology and chronic health evaluation score

**$DATA** Sitafloxacin.csv ; Data input file

IGNORE=@ ; Ignore non-data rows

**$SUBROUTINE** ADVAN5 TRANS1 ; Specify subroutine

**$MODEL** NCOMP=4

COMP=(1) ; Gut compartment

COMP=(2) ; Blood compartment

COMP=(3) ; Transit compartment 1

COMP=(4) ; ELF compartment

**$PK**

;;; F1AGE-DEFINITION START

F1AGE = ( 1 + THETA(8)*(AGE - 57))

;;; F1AGE-DEFINITION END

;;; F1-RELATION START

F1COV=F1AGE

;;; F1-RELATION END

**;;; Disposition parameters**

TVCLBD = THETA(1)*((WT/52)**0.75) ; Pop typical clearance in blood (L/hr)

CLBD = TVCLBD *EXP(ETA(1)) ; Individual clearance in blood (L/hr)

TVVBD = THETA(2)*(WT/52) ; Pop typical Vd in blood (L)

VBD = TVVBD *EXP(ETA(2)) ; Individual Vd in blood (L)

TVF1 = THETA(3) ; Population typical bioavailability

TVF1 = F1COV*TVF1

F1 = TVF1 *EXP(ETA(3)) ; Individual bioavailability

TVMTT = THETA(4) ; Typical value of mean transit time (hr)

MTT = TVMTT*EXP(ETA(4))

TVQELF = THETA(5)*((WT/52)**0.75) ; Pop typical inter-compartment CL (L/hr)

QELF = TVQELF *EXP(ETA(5)) ; Individual inter-compartment CL (L/hr)

TVVELF = THETA(6) ; Population typical Vd in ELF (L)

VELF = TVVELF *EXP(ETA(6)) ; Individual Vd in ELF (L)

TVPC = THETA(7) ; Population partition coefficient

PC = TVPC *EXP(ETA(7)) ; Individual partition coefficient

NN = 1 ; Number of transit compartments

KTR = (NN+1)/MTT ; Transit compartment rate constant

K13 = KTR

K32 = KTR

K24 = QELF*0.63*PC/VBD

K42 = QELF/VELF

K20 = CLBD/VBD ; Elimination rate constant (hr^-1^)

;;; Half-life calculation

HALF = 0.693/K20 ; Half-life (hr)

**$ERROR**

IF(CMT.EQ.2) IPRED = A(2)/VBD ; Sitafloxacin plasma conc. (ug/L)

IF(CMT.EQ.3) IPRED = A(4)/VELF ; Sitafloxacin ELF concentration (ug/L)

IF(IPRED.GT.0) IPRED = LOG(IPRED) ; Natural log the data

IF(CMT.EQ.2) THEN

W = SQRT(SIGMA(1,1))

Y = IPRED + EPS(1)

ENDIF

IF(CMT.EQ.3) THEN

W = SQRT(SIGMA(2,2))

Y = IPRED + EPS(2)

ENDIF

IRES = DV-IPRED

IWRES = IRES/W ; Individual weighted residual

**; Initial estimates Theta and Omega**

**$THETA**

(0, 7.01) ; 1. CL of blood compartment

(0, 115) ; 2. Vd of blood compartment

(1) FIX ; 3. Bioavailability

(0, 1.49) ; 4. Mean transit time

(0, 0.0714) ; 5. Intercompartment CL of ELF compartment

(0.025) FIX ; 6. Vd of ELF compartment

(0, 0.733) ; 7. Partition coefficient

(-0.056, 0.0258,0.032) ; 8. Covariate - AGE on F

**$OMEGA**

0.576 ; 1. IIV CL of blood compartment

0 FIX ; 2. IIV Vd of blood compartment

0.0274 ; 3. IIV bioavailability

0.623 ; 4. IIV mean transit time

0 FIX ; 5. IIV intercompartment CL of ELF compartment

0 FIX ; 6. IIV Vd of ELF compartment

0.0534 ; 7. IIV partition coefficient

**$SIGMA**

0.122 ; Prop_RUV of blood conc.

0.274 ; Prop_RUV of ELF conc.

**$ESTIMATION** MAXEVAL=9999 ; Number of evaluations

PRINT=5 ; Print every 5th iteration

METHOD=1 ; Estimation method: 1=First Order Conditional (FOCE)

INTER ; Estimatation with interaction between ETA's and EPS's

**$COVARIANCE** PRINT=E ; Estimating the covariance matrix (eigenvalues)

**;UNCONDITIONAL** ; Enables covariance step despite warnings

$TABLE ID TIME IPRED IWRES CWRES NPDE CMT F1 CLBD VBD MTT MDV EVID HALF NOPRINT ONEHEADER FILE=mytabRun56

$TABLE ID TIME EVID MDV IPRED IWRES CWRES IPRED CMT NOPRINT ONEHEADER FILE=sdtabRun56

$TABLE ID CLBD VBD MTT K20 ETA1 ETA2 ETA3 ETA4 CMT NOPRINT ONEHEADER FILE=patabRun56

$TABLE ID CMT SEX NOPRINT ONEHEADER FILE=catabRun56

$TABLE ID AGE WT CLCR APACHE NOPRINT ONEHEADER FILE=cotabRun56

**Code for simulation**

**$PROBLEM** Run59

;; 1-compartment disposition

;; 1 transit compartment (KA=KTR)

;; Add allometric function on QELF

;; Body weight as allometric function (median body weight)

**$INPUT** ID ; Virtual patient ID

TIME ; Hour

DV ; Natural logarithm conc. (ug/L)

AMT ; Sitafloxacin dose (ug)

CMT ; Compartment (1=Depot, 2=Blood, 3=ELF)

ADDL ; Additional dose data item

II ; Dosing interval (hours)

MDV ; Missing dependent variable: MDV=0 (DV); or MDV=1 (Dose)

EVID ; Event ID 0=Sample; Dose, 1= Dose

AGE ; Age (year)

WT ; Body weight (kg)

**$DATA**  Sitafloxacin_sim50MG.csv ; Data input file

IGNORE=@ ; Ignore non-data rows

**$ABBREVIATED COMRES=8**

**$SUBROUTINE** ADVAN13 TOL=6 ; Specify subroutine

**$MODEL** NCOMP=6

COMP=(1) ; Gut compartment

COMP=(2) ; Blood compartment

COMP=(3) ; Transit compartment 1

COMP=(4) ; ELF compartment

COMP=(5) ; Plasma sitafloxacin AUC

COMP=(6) ; ELF sitafloxacin AUC

**$PK**

;;; F1AGE-DEFINITION START

F1AGE = ( 1 + THETA(8)*(AGE - 57))

;;; F1AGE-DEFINITION END

;;; F1-RELATION START

F1COV=F1AGE

;;; F1-RELATION END

**;; Disposition parameters**

TVCLBD = THETA(1)*((WT/52)**0.75) ; Pop typical clearance in blood (L/hr)

CLBD = TVCLBD *EXP(ETA(1)) ; Individual clearance in blood (L/hr)

TVVBD = THETA(2)*(WT/52) ; Pop typical Vd in blood (L)

VBD = TVVBD *EXP(ETA(2)) ; Individual Vd in blood (L)

TVF1 = THETA(3) ; Population typical bioavailability

TVF1 = F1COV*TVF1

F1 = TVF1 *EXP(ETA(3)) ; Individual bioavailability

TVMTT = THETA(4) ; Typical value of mean transit time (hr)

MTT = TVMTT*EXP(ETA(4))

TVQELF = THETA(5)*((WT/52)**0.75) ; Pop typical inter-compartment CL (L/hr)

QELF = TVQELF *EXP(ETA(5)) ; Individual inter-compartment CL (L/hr)

TVVELF = THETA(6) ; Pop typical Vd in ELF (L)

VELF = TVVELF *EXP(ETA(6)) ; Individual Vd in ELF (L)

TVPC = THETA(7) ; Population partition coefficient

PC = TVPC *EXP(ETA(7)) ; Individual partition coefficient

NN = 1 ; Number of transit compartments

KTR = (NN+1)/MTT ; Transit compartment rate constant

K13 = KTR

K32 = KTR

K24 = QELF*0.63*PC/VBD

K42 = QELF/VELF

K20 = CLBD/VBD ; Elimination rate constant (hr^-1^)

;; Half-life calculation

HALF = 0.693/K20 ; Half-life (hr)

;;For secondary Parameters

IF(NEWIND.LE.1) THEN ; Assign negative Cmax Tmax for the new ID

COM(1)=-1 ; Holder of Cmax plasma

COM(2)=-1 ; Holder of Tmax plasma

COM(3)=-1 ; Holder of Cmax ELF

COM(4)=-1 ; Holder of Tmax ELF

COM(5)=-1 ; Holder of AUC plasma

COM(6)=-1 ; Holder of AUC plasma

COM(7)=-1 ; Holder of AUC ELF

COM(8)=-1 ; Holder of AUC ELF

ENDIF

**$DES**

DADT (1) = -A(1)*K13

DADT (2) = A(3)*K32 + A(4)*K42 - A(2)*K24 - A(2)*K20

DADT (3) = A(1)*K13 - A(3)*K32

DADT (4) = A(2)*K24 - A(4)*K42

DADT (5) = A(2)

DADT (6) = A(4)

CT1 = A(2)/VBD

CT2 = A(4)/VELF

IF(CT1.GT.COM(1)) THEN

COM(1)=CT1 ; Plasma Cmax

COM(2)=T ; Plasma Tmax

ENDIF

IF(CT2.GT.COM(3)) THEN

COM(3)=CT2 ; ELF Cmax

COM(4)=T ; ELF Tmax

ENDIF

**$ERROR**

CMAXPL = COM(1)

TMAXPL = COM(2)

CMAXELF = COM(3)

TMAXELF = COM(4)

AUCPL = A(5)/VBD

AUCELF = A(6)/VELF

IF(TIME.EQ.168.AND.CMT.EQ.2) THEN

COM(5) = AUCPL

ENDIF

IF(TIME.EQ.192.AND.CMT.EQ.2) THEN

COM(6) = AUCPL

ENDIF

IF(TIME.EQ.168.AND.CMT.EQ.3) THEN

COM(7)= AUCELF

ENDIF

IF(TIME.EQ.192.AND.CMT.EQ.3) THEN

COM(8)=AUCELF

ENDIF

AUCPL168 = COM(5)

AUCPL192 = COM(6)

AUCPL24 = AUCPL192-AUCPL168

AUCELF168 = COM(7)

AUCELF192 = COM(8)

AUCELF24 = AUCELF192-AUCELF168

IF(CMT.EQ.2) IPRED = A(5)/VBD ; Sitafloxacin plasma conc. (ug/L)

IF(CMT.EQ.3) IPRED = A(6)/VELF ; Sitafloxacin ELF conc. (ug/L)

IF(IPRED.GT.0) IPRED = LOG(IPRED) ; Natural log conc.

IF(CMT.EQ.2) THEN

W = SQRT(SIGMA(1,1))

Y = IPRED + EPS(1)

ENDIF

IF(CMT.EQ.3) THEN

W = SQRT(SIGMA(2,2))

Y = IPRED + EPS(2)

ENDIF

IRES = DV-IPRED

IWRES = IRES/W

**; Initial estimates Theta and Omega**

**$THETA**

(0, 7.03) ; 1. CL of blood compartment

(0, 116) ; 2. Vd of blood compartment

(1) FIX ; 3. Bioavailability

(0, 1.48) ; 4. Mean transit time

(0, 0.0441) ; 5. Intercompartment CL of ELF compartment

(0.025) FIX ; 6. Vd of ELF compartment

(0, 0.772) ; 7. Partition coefficient

(-0.056, 0.0258,0.032) ; 8. Covariate - AGE on F

**$OMEGA**

0.566 ; 1. IIV CL of blood compartment

0 FIX ; 2. IIV Vd of blood compartment

0.0285 ; 3. IIV bioavailability

0.626 ; 4. IIV mean transit time

0 FIX ; 5. IIV intercompartment CL of ELF compartment

0 FIX ; 6. IIV Vd of ELF compartment

0.0528 ; 7. IIV partition coefficient

**$SIGMA**

0.122 ; Prop_RUV of blood conc.

0.249 ; Prop_RUV of ELF conc.

**$SIMULATION** (2825054) ONLYSIM SUBPROBLEMS=1000

**;UNCONDITIONAL** ; Enables covariance step despite warnings

$TABLE ID TIME IPRED IWRES CWRES NPDE CMT F1 CLBD VBD MTT AUCPL192 AUCPL168 AUCPL24 AUCELF192 AUCELF168 AUCELF24 MDV EVID HALF CMAXPL TMAXPL CMAXELF TMAXELF NOPRINT ONEHEADER FILE=mytabRun59

;$TABLE ID TIME EVID MDV IPRED IWRES CWRES IPRED CMT NOPRINT ONEHEADER FILE=sdtabRun59

;$TABLE ID CLBD VBD MTT K20 ETA1 ETA2 ETA3 ETA4 CMT NOPRINT ONEHEADER FILE=patabRun59

;$TABLE ID CMT SEX NOPRINT ONEHEADER FILE=catabRun59

;$TABLE ID AGE WT CLCR APACHE NOPRINT ONEHEADER FILE=cotabRun59
